# Editorial: Case reports in aging psychiatry

**DOI:** 10.3389/fpsyt.2023.1306422

**Published:** 2023-11-28

**Authors:** Ram J. Bishnoi

**Affiliations:** Department of Psychiatry and Behavioral Neurosciences, University of South Florida, Tampa, FL, United States

**Keywords:** case reports (publication type), aging, dementia, neurocognitive disorder, atypical presentation

## Introduction

Case reports are an important part of scientific literature, where exceptional or novel situations, for which there exist no clear or deterministic data or evidence, are presented. These reports have immense educational value, and at times, they propose new hypotheses for further empirical evidence generation (1). In this special section of Aging Psychiatry, unusual presentations of dementing disorders, and their clinical expressions, are described from an etiopathological point of view.

## Autoimmune dementia

Neural antibodies have been reported to cause neuroinflammation and, therefore, cognitive impairment. Hansen, Sagebiel et al. from the University of Goettingen, Germany, report three distinct cases of dementia syndrome associated with neural antibodies. The identification of these types of patients can have significant treatment implications and requires further research to characterize these patients for clinical identification. In a series of three cases, the first case describes the presence of autoantibodies toward VGlut2 (vesicular glutamate transporter 2) in addition to Alzheimer's pathology (Hansen, Teegen, Hirschel, Willfang, Schott, Bartels et al.).

Subsequently, a 75-year-old patient diagnosed with dementia exhibited moderate cerebral microangiopathy upon neuroimaging and tested positive for anti-CARPVIII autoantibodies during serum analysis. In light of both cerebrovascular alterations and the presence of neuroinflammation, the patient was diagnosed with mixed dementia, encompassing both vascular and autoimmune components (Hansen, Teegen, Hirschel, Willfang, Schott, Malchow et al.).

Finally, Hansen, Sagebiel et al. describe two patients who presented with mild cognitive impairment, and brain MRI showed microangiopathy. Neurofascin 186 antibodies were detected in serum in both patients. These cases highlight that neurofascin 186 antibodies not only cause peripheral neuropathy but can also induce central neuroinflammation. These cases add to the expanding literature on autoimmune dementia and argue for more research in this direction.

## Unusual presentation of Lewy body dementia

Lewy body dementia (LBD) is the second most common neurodegenerative dementia, and its diagnosis, especially in early stages, can be challenging. Depression is a common comorbidity with LBD. The question of whether late-onset depression serves as a risk factor or a prodrome of dementia remains to be answered (2). Valença et al. present a case of a 73-year-old patient who was treated for refractory depression for 3 years while his cognitive functions deteriorated. The patient was eventually found to have biomarkers that confirmed LBD. This case highlights the importance of a clear understanding of the depression–dementia relationship.

In another noteworthy case series on LBD, Taomoto et al. reported two cases in which patients exhibited clinical symptoms of LBD accompanied by delusions of infestation. While hallucinations, particularly visual ones, are well-established features of LBD, this case series underscores the significance of recognizing tactile hallucinations as potential symptoms of LBD. Furthermore, it highlights the potential efficacy of acetylcholinesterase inhibitors in mitigating these distressing symptoms.

## Unusual presentation of encephalitis

Porpiglia et al. present an unusual clinical presentation of anti-leucine-rich glioma-inactivated 1 (LGI1) encephalitis, a type of limbic encephalitis, which presented as a manic episode with cognitive symptoms. Treatment with intravenous immunoglobulins led to improvement of neuropsychiatric symptoms and corresponding changes in functional neuroimaging. This case not only serves to inform us about this unique and atypical presentation but also emphasizes the significance of identifying warning signs for the diagnosis. Additionally, complete recovery of this patient with immune system modulators provides a direction for future treatment research.

In summary, these cases present unique clinical scenarios that may not typically be considered during a routine clinical examination and treatment planning process. Moreover, the collection of such case studies provides a framework for initial data collection, eventually leading to newer conceptual categories. For example, autoimmune dementia and encephalopathies can present with a variety of symptoms as described by Hansen, Teegen, Hirschel, Willfang, Schott, Malchow et al.. These case reports prompt consideration of autoimmune phenomena in similar clinical presentations and propose a new research context. Research findings and learning from this area can have implications for more common neurocognitive disorders secondary to neurodegenerative and cerebrovascular disorders.

## Author contributions

RB: Writing—original draft, Writing—review & editing.
